# Implementation of a nursing strategy for self-transcendence in older adults: an experience report

**DOI:** 10.1590/0034-7167-2022-0745

**Published:** 2023-11-27

**Authors:** Jesús Guadalupe Martínez Ramírez, Erika Adriana Torres Hernández, Raúl Fernando Guerrero-Castañeda

**Affiliations:** I Universidad de Guanajuato. Celaya, Guanajuato, Mexico; II Universidad Autónoma de San Luis Potosí. San Luis Potosí, San Luis Potosí, Mexico

**Keywords:** Nurses, Nursing Theory, Nurse-Patient Relations, Aged, Spirituality, Enfermeiras e Enfermeiros, Teoria de Enfermagem, Relações Enfermeiro-Paciente, Idoso, Espiritualidade, Enfermeras y Enfermeros, Teoría de Enfermería, Relaciones Enfermero-Paciente, Anciano, Espiritualidad

## Abstract

**Objectives:**

to report the experience of implementing a nursing strategy to promote self-transcendence in older adults attended at a Primary Health Care center.

**Methods:**

an experience report on the implementation of a strategy addressing the Theory of Self-Transcendence by nursing to older adults in a primary health unit, in San Luis Potosí, Mexico, from October to November 2022.

**Results:**

fourteen older adults and three nurses participated. From the expression of feelings, speeches about self-transcendence, spirituality and physical activity promotion, the strategy was satisfactory. Emotional, cognitive and spiritual changes were observed in the different dimensions of the Theory of Self-Transcendence.

**Final Considerations:**

the nursing strategy on self-transcendence made it possible to identify significant moments that helped to identify the key points in which older adults were; it helped to intervene from a person-to-person care perspective.

## RESUMEN

**Objetivos:**

relatar la experiencia de la implementación de una estrategia de enfermería para promover la autotrascendencia en adultos mayores que acuden a un centro de primer nivel de atención en salud.

**Métodos:**

relato de experiencia sobre implementación de una estrategia abordando la Teoría de Autotrascendencia por enfermería a adultos mayores de una unidad de salud de primer nivel, en San Luis Potosí, México, de octubre-noviembre del 2022.

**Resultados:**

participaron 14 adultos mayores y tres enfermeros. A partir de la expresión de sentimientos, charlas sobre autotrascendencia, fomento de espiritualidad y actividad física, la estrategia resultó satisfactoria. Se observaron cambios emocionales, cognitivos y espirituales en las diferentes dimensiones de la Teoría de Autotrascendencia.

**Consideraciones Finales:**

la estrategia de enfermería sobre autotrascendencia permitió plasmar momentos significativos que ayudaron a identificar puntos clave en los que se encontraban los adultos mayores; esto ayudó para intervenir desde una perspectiva del cuidado de persona a persona.

## RESUMO

**Objetivos:**

relatar a experiência da implementação de uma estratégia de enfermagem para promover a autotranscendência em idosos atendidos em um centro de Atenção Primária À Saúde.

**Métodos:**

relato de experiência sobre a implementação de uma estratégia abordando a Teoria da Autotranscendência pela enfermagem a idosos de uma unidade de saúde de primária, em San Luis Potosí, México, de outubro a novembro de 2022.

**Resultados:**

participaram 14 idosos e três enfermeiros. A partir da expressão de sentimentos, falas sobre autotranscendência, promoção da espiritualidade e atividade física, a estratégia foi satisfatória, e foram observadas mudanças emocionais, cognitivas e espirituais nas diferentes dimensões da Teoria da Autotranscendência.

**Considerações Finais:**

a estratégia de enfermagem sobre autotranscendência possibilitou identificar momentos significativos que ajudaram a identificar os pontos-chave em que os idosos se encontravam; isso ajudou a intervir a partir de uma perspectiva de cuidado de pessoa para pessoa.

## INTRODUCTION

When talking about self-transcendence, reference is made to the ability to expand one’s limits in various ways that improve well-being. These boundaries can be expanded intrapersonally, interpersonally, transpersonally, and temporally^(1)^. To transcend, older adults must explore their ability to exist^(1)^; in other words, they must face any context and/or situation that compromises their health, be it physical, emotional, psychological or spiritual.

Self-transcendence implies spiritual issues that comprise the highest human values, such as love, compassion and respect for life with oneself and the environment, identifying the problems and realities that can positively influence older adults’ physical and mental health^(2)^.

On the other hand, it has been demonstrated that the self-transcendence process has effects on one’s own well-being and is a predictor of a greater perception of quality of life, in relation to older adults; a process that increases self-awareness, self-encounter and life satisfaction^(3)^.

Therefore, addressing self-transcendence creates a link with spirituality, both aimed at covering and satisfying their needs in all spheres, whether emotional, cognitive or spiritual.

The Theory of Self-Transcendence implementation in a Primary Health Care institution is a priority and has social and disciplinary relevance for nursing, since it is the first approach and contact with older adults. The primary level favors health and education promotion, which favors well-being. This theory addresses the concept of well-being as a balance point, with nursing being responsible for promoting self-transcendence at inter, intra and transpersonal levels^(1)^. Therefore, promoting health and well-being at this level would be fundamental for the development of older adults. Thus, the nursing team, by fostering the nurse-caregiver bond, generates trust and empathy, allowing them to feel heard and valued by a health professional^(2,4)^.

Therefore, this document reports the experience of professional practice when showing the analysis in the light of Pamela G Reed’s Theory of Self-Transcendence, contemplating the proposed dimensions, vulnerability, self-transcendence and well-being as well as the spirituality domains in old age. Through this experience report, a situation is presented that allows the nursing team to observe, describe and obtain information from the issuing population, in order to achieve a holistic and professional view of nursing care and, subsequently, tell that story.

## OBJECTIVES

To report the experience of implementing a nursing strategy to promote self-transcendence in older adults assisted at a Primary Health Care center.

## METHODS

This is an experience report of professional nursing practice on the approach of the Theory of Self-Transcendence in older adults assisted in a health unit in the city of San Luis Potosí, capital. This health unit is of a Primary Health Care unit, and activities are carried out aimed at older adults, such as general consultations (assessment), lectures on any topic related to health, physical activity and handicrafts.

To achieve the above, a health education program was designed (Chart 1) under the approach of the Theory of Self-Transcendence. Activities were located that allowed the approach of each of the dimensions of the theory: vulnerability, self-transcendence and well-being. From October to November, workshops and lectures were held in six sessions (two of them divided into 2 parts) (one session per week, lasting 60 minutes), with the participation of 14 older adults, a head nurse who developed the workshop and two medical nurses who contributed to design and assessment and also supervised implementation. The workshop started with an integration dynamic, followed by a presentation supported by slides by a nurse. At the end of presentation, older adults were asked to express their feelings and emotions regarding the subject addressed; finally, the resolution of doubts on the subject and the closure of the session with the contribution of each older adult about the workshop/lecture to assess how older adult went back home.

**Chart 1:** Dimensions addressed by the Theory of Self-Transcendence

Vulnerability dimensions		
**Dimension description**	**Objective**	**Activities**
It refers to the awareness of mortality that a person has. This dimension is addressed from the diagnosed diseases of older adults.	Know the generalities of older adults as well as chronic-degenerative diseases, disorders, pathologies among others.	- Generate a serene environment with older adults. - Motivate older adults to express their feelings and emotions. - Encourage active listening by nurses. - Establish effective communication.
**Self-transcendence dimension**		
**Dimension description**	**Objective**	**Activities**
It refers to the ability to expand one’s limits in different ways that enhance well-being. This dimension is approached from self-conception and giving meaning to the present, facing any context and/or life situation.	Identify the needs to transcend oneself as well as promote a source beyond oneself.	- Tell the experiences about their own needs. - Give a talk on the subject of self-transcendence. - Create a link between caregiver and nurse. - Encourage creative problem solving.
**Well-being dimension**		
**Dimension description**	**Objective**	**Activities**
It is a subjective feeling of health or fullness based on each person’s own criteria at a given time. This dimension is addressed from the process of self-transcendence of each older adult to reach its total plenitude.	Contribute to the well-being that older adults need, involving self-sufficiency to be happy, healthy and satisfied in their life journey.	- Present a talk with clear and understandable language about spirituality, well-being and functionality. - Carry out a spiritual reading by nurses in a group to older adults to reflect on this dimension. - Help the expression of feelings and emotions. - Encourage the performance of physical activities, stretching and muscle warm-up exercises, breathing exercises, exercises for coping with stress, among other activities. - Share speeches about what has been learned and promoted.

The health education program was approved to be carried out by authorities of the Comprehensive Care and Health Research Unit (CCHRU) of the Faculty of Nursing and Nutrition at the *Universidad Autónoma de San Luis Potosí*. It complied with national and international ethical guidelines, since it is not a research study, submitting it to a Research Ethics Committee for approval was waived. However, the ethical considerations of integrity and respect for participant anonymity were respected in the case of this experience report. The consent signed by participants was waived, since it is not a research study; however, the criteria of anonymity, confidentiality and respect for older adult participants’ integrity were met.

## RESULTS

### 1^st^ session: meeting the older adult group

As the first session, a nurse appeared at the health unit, which is a Primary Health Care center, located in the city of San Luis Potosí, capital. There was an approach to know the generalities of the group and who accompanied older adults to this center. This first approach made it possible to meet the group members, with older adults being informed that they would participate with them in a series of workshops and lectures on strategies that would help them to feel good about themselves and, therefore, would provide feedback to nurses, as a care professional, to find the best intervention strategies in future sessions. It is noteworthy that this activity caught their attention, and they agreed to participate in scheduled sessions.

Regarding the older adults who attend the health unit, most like to talk, walk outside, do physical activity, take care of their grandchildren, support their children, but they like to attend the unit more, because they can live together and interact with other older adults. Some live and live with their family, others live with their family but don’t live together, and some do not live with anyone.

Those who have children bring their grandchildren to take care of them and live with them; those who have children, but do not visit them, go out for a walk to unwind and talk to other people outside their social circle; those who live alone look for different ways to feel accompanied (go for a walk, talk to the neighbors, do housewife activities to keep busy and make the most of their time).

Of the same adult population, the smallest part has chronic-degenerative diseases (diabetes and high blood pressure), psychiatric diseases (schizophrenia), limited functionality (paraplegia), and most older adults consider themselves to have good health (healthy).

### 2^nd^ session: dynamic eyes closed and covered to express feelings

From the 1st session, the objective of identifying the contextual/social elements and thus strengthening self-transcendence in the following sessions was resumed. It began with the session entitled “aging and self-transcendence”: a nurse introduced himself to the older adult in the group and explained the objectives. The first strategy was to tell the experiences about older adults’ emotional needs: the older adult population was advised that it would start with a dynamic, and they were given a bandana to cover their eyes, then indications were mentioned.

The dynamics were as follows: a calm environment was created and a bandana was placed over the eyes; once covered, they were randomly passed to the locations of each drawn older adult; given a small ball at random; they expressed and expressed how they were today, listening to what they express and feel, since each older adult expressed their feelings and emotions; continued to ask other seniors once they had finished expressing their feelings and emotions; they were instructed to remove the bandana from their eyes.

The expressions that older adults had at that moment were directed to feelings of “happiness”, such as:

*At this moment, I am happy, because we are alive.* (Older adult 1)

*I am happy, because I have no longer lost control of my diabetes.* (Older adult 2)

Other expressions were related to “sadness”, such as:

*I feel sad, because my son has not brought my grandchildren home.* (Older adult 3)

### 3^rd^ session: talking about self-transcendence

The second activity was self-transcendence presentation: a PowerPoint presentation begins in a specific room of the health unit; it was explained to them in a simple, understandable and clear language in which older adults can specifically understand and leave with a meaningful education and learning; if there were any doubts, they could interrupt without questioning so that it would be clear to them.

Once the talk was over, older adults were asked for their point of view if it was good, bad, boring, among others, to which some responded:

*You just helped me on how to learn to make good decisions.* (Older adult 4)

*We must learn to leave a mark on our family.* (Older adult 5)

So, according to their comments, there was no doubt, and they were able to understand the context of self-transcendence. They were provided with the necessary tools so that they can further extend their ability to love and value themselves even more, such as inhale the air through the nose and exhale it through the mouth, and do it a couple of times, where to turn when they feel sad, lonely, how to start a conversation with people of different ages, and that they should be their own motivation.

### 4^th^ session: learning to grow old being spiritual

After knowing its context and the social, the following session entitled “always spiritual” was held. Since older adults already knew the nurses, they immediately went on to scheduled activities. The first activity was to express: What do you use to feel good?

The dynamic was that each one expressed and externalized what mechanisms they resort to, when they are in a bad moment so that they can feel better. The others continued to be asked consecutively, once they had finished expressing the mechanisms, then they began with the corresponding presentation. The expressions that older adults had were focused on “expressing what they feel”:

*Well, the truth is I just say things and I don’t try to keep anything. Carry out relaxation activities.* (Older adult 6)

*I try to breathe so as not to give it the necessary importance, and it does not affect my health.* (Older adult 7)

*I go for a walk to clear my mind.* (Older adult 8)

And, finally, some older adults resort to “something religious”, such as:

*I start doing what I like, be it praying, knitting and cooking.* (Older adult 9)

*I start to pray for God to take care of me and protect me.* (Older adult 10)

### 5^th^ session: fostering spirituality

The next activity was the presentation on spirituality: starting presentation, there was a lecture in clear and understandable language so that they could learn how to promote spirituality and how they can properly accept the phase of old age and strengthen their own well-being. At the end of the lecture, a nurse held a group spiritual reading for older adults to reflect on this dimension; later, they expressed what they felt at that moment and, finally, they performed breathing exercises and expressed aloud what they wanted to release. Some commented:

*Problems I leave them on the ground and I don’t want them to torment me anymore* [jumps on the ground, and comments that he already stepped on them and won’t carry them again]. (Older adult 11)

*God give me strength to get ahead and continue as up to now as a strong woman.* (Older adult 12)

Based on these comments, older adults reflected on the spiritual, and a very significant phrase was “let’s search for more years of life”. They were able to understand the spirituality process, and the tools provided were helpful in expressing their emotions and, most importantly, looking after their own well-being.

### 6^th^ session: being active for my health and enjoying music

After addressing the spiritual dimension, the workshop/lecture “moving for my health” began, and the scheduled activities were immediately addressed. As the first activity was to provide a sheet of paper to each older adult, they were informed that they would follow the instructions to assess the folds on the sheet. These exercises helped in older adults’ coordination and learning, correctly following each of the indications.

It was identified that older adults still have good motor skills, coordination at the extremities and a very adequate facility to make each fold, and they still did it with such impressive delicacy that they were motivated to make each fold more directional.

At the end of this activity, older adults were invited to the health unit’s courtyard, where they performed physical activities, being highly motivated by *Cumbia*. They started with stretching exercises for 10 minutes, then expressed that they felt superactive and that they wanted to increase exercise intensity, which met this need.

To finish the physical activity, they practiced the breathing exercises already learned in the previous sessions so that they left calm and relaxed, and some expressed phrases aimed at “well-being”:

*I put into practice everything he taught me and my family too, I am grateful because now they pay more attention to me and I feel much better.* (Older adult 13)

*In part, I feel very good about myself and I also try to socialize with more people around me.* (Older adult 14)

These comments marked a lot, a lot to learn, and they fill with peace and joy to know that everything that was addressed in workshops is working for them to improve their quality of life, mainly to be more functional and able to self-transcend on their own and most importantly they know the importance of moving and being physically active.

## DISCUSSION

The sessions of this workshop/lecture were useful, as the self-transcendence process was applied, and it can be seen that each of the dimensions of this Theory of Self-Transcendence were covered and, therefore, satisfactory for each of the older adults.

The *vulnerability* dimension according to Reed refers to the awareness of mortality that a person has, i.e., a variety of human experiences that increase this awareness, but are health-related events that endanger life or imply a loss^(1)^. Within this dimension, loneliness was found in older adults, some with chronic degenerative diseases (diabetes and arterial hypertension), psychiatric diseases (schizophrenia), limited functionality (paraplegia). For this, interventions were carried out that contemplated this dimension such as: generating a calm and serene environment^(5)^; helping older adults to express their feelings and emotions^(6)^; performing active listening by nurses^(2)^; and making effective communication (understandable language)^(4)^. It has been demonstrated that older adults with a chronic disease face challenges that weigh on a person’s health status, which leads to negative thoughts and makes them vulnerable to face a certain situation^(7)^. Within the strategy, the presence of diseases in older adults was approached from the concept of vulnerability, which can be seen as a challenging situation for older adults.

In the *self-transcendence* dimension, Reed argues that it is the ability to expand one’s limits in different ways that improve well-being, such as intrapersonal (for greater awareness of one’s beliefs, values and dreams), interpersonal (to connect with others and the environment), transpersonal (relating to dimensions beyond the observable world) and temporally (integrating past and future and making sense of the present)^(1)^. Characteristics were found here such as: older adults seek different ways to feel accompanied, accept chronic degenerative diseases and prevent them. And they feel good about themselves and are more motivated by physical activation. Nursing interventions were carried out to strengthen self-transcendence such as: creating a bond between caregivers and nurses as well as observing and analyzing the context of the situation^(8)^; generating creative problem solving (teaching-learning); performing breathing and meditation exercises, where to resort to when feel sad, alone, how to start a conversation with people of different ages, and which are their own motivation^(6,9)^; encouraging physical activities to strengthen functionality^(10)^. Finally, in the *well-being* dimension, Reed defines it as a subjective feeling of health or completeness based on a person’s own criteria at a given moment^(1)^.

It was found that older adults know what mechanisms to resort to, when they are in a bad moment to feel better. Interventions were implemented to address this dimension: talking in clear and understandable language; holding a group spiritual reading for seniors to reflect on this dimension^(2)^; and performing breathing exercises and expressed aloud what they wanted to release^(6)^. Self-transcendence allows older adults to work on the situation and go beyond their limits of understanding to reflect and thus make a clear decision^(6,9)^, adopting measures to prevent risky behavior, thus being able to determine actions that lead to their own well-being^(7)^. The ways of understanding their reality were evidenced from the activities carried out with spirituality as a mediator^(1)^ as well as the physical activity that enhances these factors.

In the authors’ experience, addressing these sessions allowed us to come to light of Pamela G Reed’s Theory of Self-Transcendence; However, this issue is considered little addressed by the nursing staff, mainly in the older adult population. People being cared for (older adults) were open to communication, dialogue and, most importantly, to reflect on their own experiences about loving, valuing, socializing, and being able to connect with the afterlife. These sessions contributed very important data and characteristics to this story. On the other hand, older adults reported feeling listened to, valued, important, happy, attentive and very active in physical activities, describing the environment in which sessions and activities were carried out as pleasant and serene. This process was building their self-esteem, feeling good spiritually and being motivated by physical activation, throughout the sessions addressed, and it was pleasant to know their experiences, feelings and emotions for which older adults were studying.

### Study limitations

The present health education program had no substantial limitations, and older adults were well received and attended in all sessions. Perhaps a limitation could be the initial development of the proposal, which was corrected when it was reviewed by experts in the selected theory and in the development of educational health programs.

### Contributions to nursing

The experience of addressing self-transcendence in older adults was very satisfactory. A great challenge was to plan on the didactic sequences from day to day on the journey that were applied in this workshop. This self-transcending process can be applied in any field of health, be it primary, secondary or tertiary level of care. Not necessarily to address the process of self-transcendence it is necessary to give lectures, workshops, talks, among others, but it can be applied at all times, because they are nursing care that, when approached, allow a mutual interaction between the people being cared for and caregivers.

It also seeks to generate and contribute knowledge about self-transcendence to reflect on and guide nursing care practice and meet the needs expressed by older adults.

## FINAL CONSIDERATIONS

This experience report clarifies the conduct of more studies on the approach of the Theory of Self-Transcendence in older adults, where more interventions can be provided according to the context in which persons in care are located, helping nurses’ professional practice to intervene not only as a professional but as a person-to-person care. This experience made it possible to create a strong bond between caregivers (older adults) and nurses, because their needs were identified and addressed so that they were mostly covered. Nursing theory helped as a preceptor to address its dimensions of vulnerability, self-transcendence and well-being, and bring them into professional practice and intervene with health education in older adults.
